# Factors Associated with Placenta Praevia in Primigravidas and Its Pregnancy Outcome

**DOI:** 10.1155/2014/270120

**Published:** 2014-11-12

**Authors:** Abdul Ghani Nur Azurah, Zakaria Wan Zainol, Pei Shan Lim, Mohd Nasir Shafiee, Nirmala Kampan, Wan Syahirah Mohsin, Norfilza Mohd Mokhtar, Muhammad Abdul Jamil Muhammad Yassin

**Affiliations:** ^1^Department of Obstetrics & Gynaecology, UKMMC, Jalan Yaacob Latiff, 56100 Kuala Lumpur, Malaysia; ^2^UKM Molecular Biology Institute, Jalan Yaacob Latiff, 56100 Kuala Lumpur, Malaysia

## Abstract

*Aim.* To examine the factors associated with placenta praevia in primigravidas and also compare the pregnancy outcomes between primigravidas and nonprimigravidas. *Method.* A retrospective cohort study was conducted in women who underwent caesarean section for major placenta praevia in a tertiary university hospital from January 2007 till December 2013. Medical records were reviewed. *Result.* Among 243 with major placenta praevia, 56 (23.0%) were primigravidas and 187 (77.0%) were nonprimigravidas. Factors associated with placenta praevia in the primigravidas were history of assisted conception (*P* = 0.02) and history of endometriosis (*P* = 0.01). For maternal outcomes, the nonprimigravidas required earlier delivery than primigravidas (35.76 ± 2.54 weeks versus 36.52 ± 1.95 weeks, *P* = 0.03) and had greater blood loss (*P* = 0.04). A vast majority of the primigravidas had either posterior type II or type III placenta praevia. As for neonatal outcomes, the Apgar score at 1 minute was significantly lower for the nonprimigravidas (7.89 ± 1.72 versus 8.39 ± 1.288.39 ± 1.28, *P* = 0.02). *Conclusion.* This study highlighted that endometriosis and assisted conception were highly associated with placenta praevia in primigravida. Understanding the pregnancy outcomes of women with placenta praevia can assist clinicians in identifying patients who are at higher risk of mortality and morbidity. Identifying potential risk factors in primigravida may assist in counseling and management of such patients.

## 1. Introduction

The incidence of placenta praevia was reported to be 0.5–1.0% from the total number of pregnancies [1]. However, this condition often requires intensive monitoring during hospitalization. In a tertiary university hospital in Kuala Lumpur, Malaysia, 4% of the total numbers of caesarean sections were performed for placenta praevia.

Placenta praevia has been well documented to be associated with adverse maternal outcomes as well as neonatal outcomes [2]. Studies have reported 5% of obstetric hysterectomies were due to placenta praevia [3, 4]. The indication for emergency peripartum hysterectomy in recent years has changed from traditional uterine atony to abnormal placentation that has now become a more common indication due to greater number of pregnant women with previous caesarean scar. Placenta praevia remains a risk factor for various maternal complications. There were higher incidence of postpartum haemorrhage (PPH) and blood transfusion in women with placenta praevia compared to general population [5–7]. Women with placenta praevia were more likely to deliver babies before 37 weeks with Apgar score of less than 7 [8]. Studies also showed that there were higher admission to neonatal intensive care unit, stillbirth and death [8, 9].

The exact pathophysiology of placenta praevia is unknown; however it has been postulated that uterine scarring may be responsible for this abnormal implantation. Adverse maternal ages, higher parity, caesarean delivery, previous curettage, history of placenta praevia, and abnormal uterus have been associated with increased risks of placenta praevia [2, 10]. Recently, Healy and colleagues reported higher incidence of placenta praevia in endometriosis patients who conceived with artificial reproductive technique as compared to patients without endometriosis [11]. To date, the occurrence of placenta praevia in primigravida without significant risk is poorly understood. It is not known whether undiagnosed endometriosis in such patients may be responsible for the occurrence of placenta praevia.

The aim of the study was to examine the pregnancy outcomes among primigravidas with major placenta praevia compared to nonprimigravidas. The study also aimed to identify the factors associated with placenta praevia in primigravida.

## 2. Material and Methods

A retrospective cohort study was undertaken to evaluate the associated factors and pregnancy outcomes in primigravidas with major placenta praevia. The inclusion criteria were all women who underwent caesarean section for major placenta praevia in the Department of Obstetrics and Gynaecology, UKM Medical Centre, from January 2007 till December 2013. The exclusion criteria were women with missing medical notes. All operative procedures performed in our centre were entered in the operative book. The books were kept at the reception area in the theatre. The hospital registration numbers of women who underwent caesarean section for major placenta praevia were obtained from the operating theatre book. Using the hospital registration number, the medical notes of these women were retrieved from the record office. The medical notes were reviewed for demographic data, intraoperative findings, and postoperative management and entered into the data sheet.

This study was approved by the ethical review board of the UKM Medical Centre, Malaysia, and funded by the Young Researcher's Grant, Universiti Kebangsaan Malaysia.

All data were analyzed using SPSS version 21.0. Data were presented as means for continuous variables and percentages for categorical variables. Continuous variables were analyzed and compared using Student's *t*-test. Categorical variables were analyzed and compared using Pearson Chi Square and Yates Continuity Corrections and *P* values of <0.05 were considered to indicate statistical significance.

## 3. Results

A total of 270 women with major placenta praevia were identified from the operating theatre book; however only 243 medical notes were available to be reviewed. Out of the total 243 women who were diagnosed with major placenta praevia, 56 (23.0%) were primigravidas and 187 (77.0%) were nonprimigravidas.

The sociodemographic data of the primigravidas and nonprimigravidas with major placenta praevia are presented in the Table 1. The primigravidas were younger and lighter than the nonprimigravidas. The ethnic population in Malaysia comprises Malays (60%), Chinese (20%), Indians (10%), and others (10%). The majority of the sample population was Malays (70%), followed by Chinese (24%), others (4%), and Indian (1%). This was similar to the Malaysian general population. There was no difference in the ethnicity, occupation, and smoking habit between the two groups.

Table 2 shows the factors associated with placenta praevia among primigravidas and nonprimigravidas. The primigravidas had significantly higher assisted conception (8.9% versus 2.1%) and history of endometriosis (21.4% versus 6.9%). A third of primigravidas (32.1%) had history of subfertility compared to only 23.5% of nonprimigravidas; however it was not statistically significant. Thirty-three percent of the nonprimigravidas gave history of previous scar and 28% had curettage performed on them.

Table 3 shows the comparison of obstetric data between the primigravidas and nonprimigravidas. The primigravidas were admitted earlier than the nonprimigravidas (31.68 ± 4.49 weeks versus 32.92 ± 3.55 weeks). There was no significant difference in the incidence of antepartum haemorrhage (APH) and receiving dexamethasone in both groups. Interestingly, none of the primigravidas had placenta located anteriorly. A vast majority of the primigravidas had either posterior type II or type III placenta praevia. The preoperative haemoglobin was similar for both groups. Only 5.8% [11] of nonprimigravidas had undergone MRI for suspected placenta accreta. Out of 11, seven women had features highly suggestive of placenta accreta on MRI.

Table 4 shows the comparison of obstetric outcomes between the primigravidas and nonprimigravidas. Two classical caesarean delivery cases were performed in the nonprimigravidas. The estimated blood loss was significantly higher in the nonprimigravidas as compared to primigravidas. Nine women (4.8%) required additional procedures being performed intraoperatively to arrest the bleeding which include six hysterectomies.. However, the postoperative haemoglobin was similar between the two groups. There was no maternal death in our sample population.

Table 5 shows the comparison of neonatal outcomes between the primigravidas and nonprimigravidas. There were more female babies being born to primigravida group. The Apgar score at 1 minute was significantly lower for the nonprimigravidas as compared to the primigravidas (7.89 ± 1.72 versus 8.39 ± 1.28). No significant difference was observed in the weight, Apgar score at 5 minutes, cord pH, NICU admission, and fetal anomaly between the two groups.

## 4. Discussion

Placenta praevia has been reported to be associated with serious maternal morbidity and mortality and also adverse neonatal outcomes [2, 7]. The exact etiology of placenta praevia still remains unknown. However, uterine scarring has been speculated as the underlying cause of placenta praevia [9]. To date, there is paucity of data on primigravidas with placenta praevia. This is the first detailed study that examined the factors associated with occurrence of placenta praevia among primigravidas and its pregnancy outcomes.

Interestingly the present study found higher incidence of assisted conception and endometriosis in primigravidas with placenta praevia. Out of 56 primigravidas, 8.9% of them conceived following clomiphene citrate, intrauterine insemination (IUI), in vitro fertilization (IVF), and intracytoplasmic sperm injection (ICSI). Several authors have reported higher prevalence of placenta praevia among women conceived following artificial reproductive technologies (ART) [12, 13]. Romundstad and colleagues reported sixfold higher risk of placenta praevia in those who underwent ART treatment compared to those who conceived spontaneously [13].

The exact pathophysiology of placenta praevia in these ART patients remained unclear. Transfer of embryos via transcervical has been postulated to be explanation for the higher occurrence of placenta praevia following IVF/ICSI. A study by Baba and colleagues reported 80% of embryos were implanted at the site of transfer [14]. There was tendency to place the embryos at the lower part of the uterine cavity as several studies have reported more favourable outcomes with lower deposition of the embryos [15].

However, a recent study has shown similar rate of placenta praevia following IVF/ICSI and gamete intrafallopian transfer (GIFT) [11]. This finding suggests that transcervical embryo transfer is less likely to be the cause of placenta praevia in ART patients.

Several researchers suggested that mechanical placement of embryos causes the release of prostaglandin, which may lead to uterine contractility [16, 17]. This could be the possible explanation for the occurrence of implantation in the lower uterine cavity thus resulting in development of placenta praevia.

A fifth of our primigravidas (21.4%) were diagnosed with endometriosis before the pregnancy compared to only 6.9% in the nonprimigravidas. Healy and colleagues reported higher incidence of placenta praevia in endometriosis patients who conceived with ART as compared to those without the disease [11]. Studies have shown women diagnosed with endometriosis have higher prevalence of antepartum haemorrhage [18, 19]. Endometriosis has been observed to alter the characteristic of endometrium. It affects the expression of various factors and markers of receptivity during the implantation window [20]. Following ovulation, progesterone plays an important role in mediating the changes in the endometrium during the secretory phase. There has been emerging evidence that suggests that endometriosis results in progesterone resistance hence affecting the placentation [21].

In the present study, estimated blood losses in nonprimigravidas were significantly higher than primigravidas. The reduced blood loss observed in the primigravidas can be attributed to the posterior localization of the placenta and also lower prevalence of placenta accreta. Furthermore, studies have shown that the ability of uterine contractions during postpartum was better in primigravidas compared to nonprimigravidas. Uterine contractions played a very important role as a protective mechanism against intraoperative blood loss [22].

Thirty-three percent of the nonprimigravidas had history of caesarean section and 28% had history of dilatation and curettage. Caesarean section and dilation and curettage were both recognized risk factors for PPH [23]. As mentioned earlier, none of the primigravidas have anteriorly located placenta. Studies have reported higher incidence of placenta accreta in those with anterior placenta as compared to posterior [24–26]. Blood loss had also been reported to be more in anteriorly located placenta as placenta was located at the site or beneath the site of incision [27].

The risk of having placenta accreta was higher in women with placenta praevia who had previous caesarean deliveries. This can be explained by the implantation of the placenta over the scar, supporting the theory that trophoblast adherence or invasion was enhanced by previous myometrial disruption [28]. In our studies, 11 (5.9%) women were suspected of having placenta accreta whereby MRIs were performed on them. Out of 11, seven women had features suggestive of placenta accreta. Among the seven with positive features, three of them were confirmed to have placenta accrete intraoperatively whereby hysterectomies were performed. One of them had embolization performed immediately after the delivery of the baby prior to hysterectomy. One woman had negative MRI feature but unfortunately intraoperatively she had placenta accreta requiring hysterectomy. Another woman who was unbooked in this hospital presented with antepartum haemorrhage at 37 weeks to casualty. No antenatal MRI was performed on her. Hysterectomy was carried out for placenta accreta. One woman required hysterectomy for uterine atony. Two other women required Bakri balloon insertion and internal iliac artery ligation for uterine atony. Other maternal outcomes were comparable between primigravidas and nonprimigravidas.

In the present study, the neonatal outcomes for both groups were not significant except for the Apgar score. The higher Apgar score at 1 minute in primigravidas can be attributed to posterior localization of the placenta. The delivery of the babies was much easier with the posterior localization of the placenta as the placenta was less likely to be cut during the delivery of the baby hence reducing the prevalence of fetal hypoxia and anaemia in primigravidas. Furthermore the caesarean sections were performed much later for the primigravidas thus lowering the prevalence of preterm babies in this group.

There are several limitations in the present study. The retrospective nature of this study cannot include certain parameters due to limited documentation and exclude potential biases. This study was conducted only in the tertiary hospital and hence the sample population does not represent the general population. The association between endometriosis and assisted conception in primigravidas with placenta praevia can only be examined by a longitudinal prospective trial.

This study highlighted an interesting finding of the location of placenta praevia in primigravidas. Considering the high prevalence of endometriosis in primigravidas with placenta praevia, further molecular study is being carried out in order to examine the association between the two. In this study, we focus on investigating the methylation status of the uPA promoter and the levels of uPA expression in the placenta and endometrial lining of placenta praevia with suspected underlying endometriosis.

## 5. Conclusion

In summary, history of assisted conception and endometriosis were found to be associated with primigravida with placenta praevia. As for maternal outcomes, the nonprimigravidas required earlier delivery and had greater blood loss. A vast majority of the primigravidas had either posterior type II or type III placenta praevia. The Apgar score at 1 minute was significantly lower for the nonprimigravidas. Understanding the pregnancy outcomes of women with placenta praevia can assist clinicians in identifying patients who are at higher risk of mortality and morbidity. Identifying potential risk factors in primigravida may assist in counseling and management of such patients.

## Figures and Tables

**Table 1:** Sociodemographic data of women with major placenta praevia.

	Primigravida *N* = 56	Nonprimigravida *N* = 187	*P* value
Age (years)	30.44 ± 3.48	33.82 ± 4.47	0.020^a^
Race (%)			0.231^b^
Malay	43 (76.8)	128 (68.5)
Chinese	12 (21.4)	47 (25.1)
Indian	0 (0.0)	2 (1.1)
Others	1 (1.8)	10 (5.3)
Weight (kg)	61.99 ± 10.28	65.41 ± 12.19	0.040^a^
Occupation (%)			0.320^b^
Housewife	11 (19.6)	54 (28.9)
Nonprofessional	23 (41.1)	75 (40.1)
Professional	22 (39.3)	58 (31.0)
Smoking (%)	0 (0.0)	2 (1.1%)	1.000^b^

^a^ *t*-test, ^b^Chi-square test.

**Table 2:** Factors associated with major placenta praevia.

	Primigravida *N* = 56	Nonprimigravida *N* = 187	*P* value
Assisted conception (%)	5 (8.9)	4 (2.1)	0.018^a^
History of caesarean (%)	0	62 (33.1)	0.000^a^
History of endometriosis (%)	12 (21.4)	13 (6.9)	0.002^a^
History of fibroid (%)	2 (3.5)	13 (7.5)	0.545^a^
History of D&C/Hysteroscopy (%)	4 (7.1)	41 (28.0)	0.012^a^
History of subfertility (%)	18 (32.1)	44 (23.5)	0.195^a^

^a^Chi-square test.

**Table 3:** Obstetric data in women with major placenta praevia.

	Primigravida *N* = 56	Nonprimigravida *N* = 187	*P* value
Gestation at admission (weeks)	31.68 ± 4.49	32.92 ± 3.55	0.033^a^
History of antepartum haemorrhage (%)	26 (46.4)	81 (43.3)	0.681^b^
Received dexamethasone (%)	19 (33.9)	77 (43.3)	0.330^b^
Type of placenta praevia (%)			0.011^b^
Posterior type II	23 (41.1)	54 (28.9)	
Posterior type III	30 (53.6)	96 (51.3)	
Anterior type III	0	12 (6.4)	
Type IV	3 (5.3)	25 (13.4)	
Pre-op MRI (%)	0	11 (5.8)	0.421^b^
Pre-op haemoglobin (gm%)	11.70 ± 0.94	11.40 ± 1.36	0.127^a^

^a^ *t*-test, ^b^Chi-square test.

**Table 4:** Obstetric outcomes in women with major placenta praevia.

	Primigravida *N* = 56	Nonprimigravida *N* = 187	*P* value
Gestation at delivery (weeks)	36.52 ± 1.95	35.76 ± 2.54	0.020^a^
Post-op haemoglobin (gm%)	10.16 ± 1.46	10.68 ± 6.86	0.575^a^
Type of caesarean (%)			1.000^b^
Lower segment	56 (100)	185 (98.9)	
Classical	0	2 (1.1)	
Caesarean (%)			0.533^b^
Elective	32 (57.1)	98 (52.4)	
Emergency	24 (42.9)	89 (47.6)	
Estimated blood loss (mls)	524.11 ± 289.98	690.16 ± 597.34	0.005^a^
Postpartum haemorrhage (%)			0.490^b^
Primary PPH	12 (21.4)	55 (29.4)	
Secondary PPH	1 (1.8)	3 (1.6)	
Received blood transfusion (%)	8 (14.3)	40 (21.4)	0.241^b^
DIVC (%)	0	8 (4.3)	0.251^b^
Placenta accreta (intraoperative) (%)	0	5 (2.6)	0.431^b^
Additional intervention (%)			
Bakri Balloon	0	1	1.000^b^
Internal iliac artery ligation	0	1	1.000^b^
B-lynch suture	0	0	
Embolization	0	1	1.000^b^
Hysterectomy	0	6	0.386^b^
Maternal death (%)	0	0	

^a^ *t*-test, ^b^Chi-square test.

**Table 5:** Neonatal outcomes of women with major placenta praevia.

	Primigravida *N* = 56	Nonprimigravida *N* = 187	*P* value
Baby weight (kg)	2.75 ± 0.52	2.67 ± 0.56	0.354^a^
Sex (%)			0.007^b^
Female	34 (60.7)	75 (40.1)	
Male	22 (39.3)	112 (59.9)	
Apgar score			
1 minute	8.39 ± 1.28	7.89 ± 1.72	0.021^a^
5 minutes	9.41 ± 1.28	9.25 ± 1.12	0.376^a^
Cord pH	7.27 ± 0.06	7.28 ± 0.09	0.364^a^
NICU admission (%)	11 (19.6)	52 (27.8)	0.221^b^
Neonatal death (%)	1 (1.8)	1 (0.5)	0.363^b^
Fetal anomaly (%)	0	3 (1.6)	0.792^b^

^a^ *t*-test, ^b^Chi-square test.
